# Supplementary material for: When face masks signal social identity: Explaining the deep face-mask divide during the COVID-19 pandemic
Source: PLoS One. 2021 Jun 10;16(6):e0253195. doi: 10.1371/journal.pone.0253195 (PMC8191909; doi:10.1371/journal.pone.0253195)
Supplement: S1 Fig — 4-standard-error bars (2 above, 2 below) to represent 95% confidence intervals. (DOCX) [file pone.0253195.s001.docx]

**Panel 1: Beliefs about partner’s cooperation**


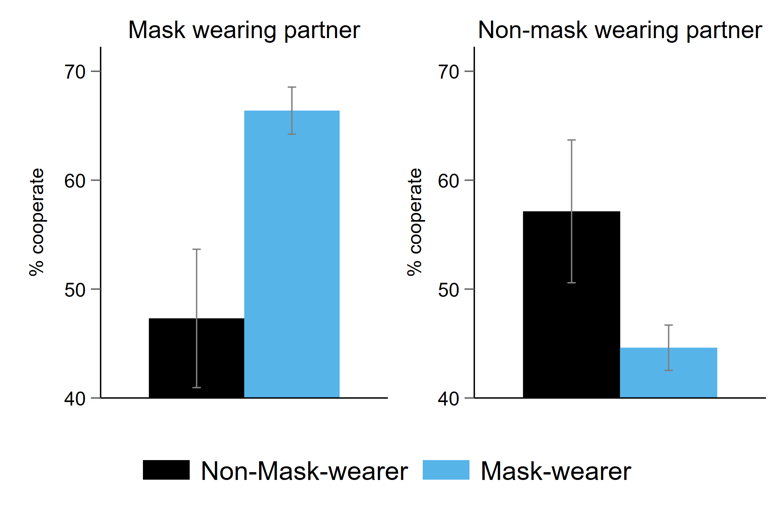


**Panel 2: Beliefs about partner’s belief of own cooperation**


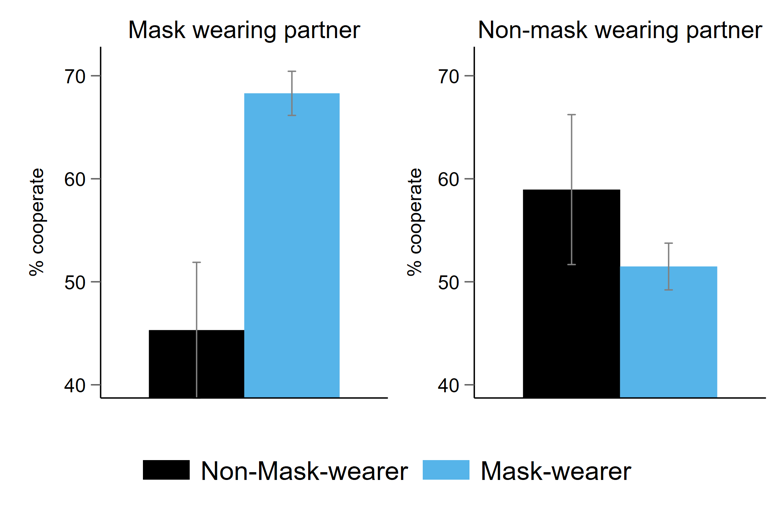


**Panel 3: Altruism towards partner**


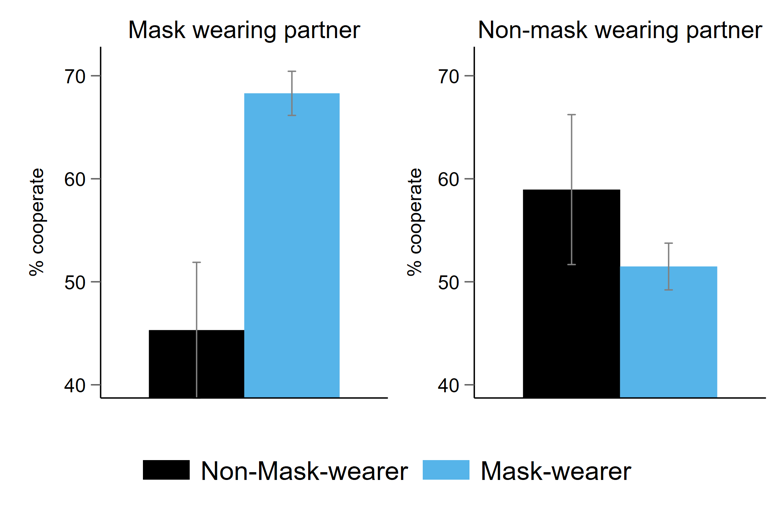


**S1 Fig: Expectations and Altruism towards Partners by mask usage.**4-standard-error bars (2 above, 2 below) to represent 95% confidence intervals.
