# Supplementary material for: When face masks signal social identity: Explaining the deep face-mask divide during the COVID-19 pandemic
Source: PLoS One. 2021 Jun 10;16(6):e0253195. doi: 10.1371/journal.pone.0253195 (PMC8191909; doi:10.1371/journal.pone.0253195)
Supplement: S1 Table — SD in parentheses. (DOCX) [file pone.0253195.s002.docx]

**S1 Table: Summary Statistics for Key Demographic Variables**

|  | Non-Mask-wearer (n=59) | Mask-wearer  (n=556) | Aggregate  (n=615) |
| --- | --- | --- | --- |
| **Age** |  |  |  |
| 20 and younger | 0.0678 | 0.0935 | 0.0911 |
|  | (0.254) | (0.291) | (0.288) |
| 20 - 29 | 0.203 | 0.338 | 0.325 |
|  | (0.406) | (0.473) | (0.469) |
| 30 - 39 | 0.356 | 0.279 | 0.286 |
|  | (0.483) | (0.449) | (0.452) |
| 40 - 49 | 0.220 | 0.149 | 0.156 |
|  | (0.418) | (0.357) | (0.363) |
| 50 - 59 | 0.102 | 0.0989 | 0.0992 |
|  | (0.305) | (0.299) | (0.299) |
| 60 and older | 0.0508 | 0.0414 | 0.0423 |
|  | (0.222) | (0.199) | (0.201) |
| *Values here reflect proportions within the corresponding population.* | | | |
| **Gender** |  |  |  |
| Female | 0.508 | 0.469 | 0.473 |
|  | (0.504) | (0.500) | (0.500) |
| Male | 0.475 | 0.522 | 0.517 |
|  | (0.504) | (0.500) | (0.500) |
| Declined to answer | 0.0169 | 0.00899 | 0.00976 |
|  | (0.130) | (0.0945) | (0.0984) |
| *Values here reflect proportions within the corresponding population.* | | | |
| **Ethnicity** |  |  |  |
| Asian/Pacific Islander | 0.0339 | 0.0899 | 0.0846 |
|  | (0.183) | (0.286) | (0.278) |
| Black/African American | 0.0169 | 0.0755 | 0.0699 |
|  | (0.130) | (0.264) | (0.255) |
| Hispanic/Latino | 0.102 | 0.0665 | 0.0699 |
|  | (0.305) | (0.249) | (0.255) |
| Native American/American Indian | 0 | 0.00180 | 0.00163 |
|  | (0) | (0.0424) | (0.0403) |
| Multiracial/Other | 0.0339 | 0.0396 | 0.0390 |
|  | (0.183) | (0.195) | (0.194) |
| White/Caucasian | 0.814 | 0.727 | 0.735 |
|  | (0.393) | (0.446) | (0.442) |
| *Values here reflect proportions within the corresponding population.* | | | |
| **Annual Household Income** |  |  |  |
| <$10,000 | 0.119 | 0.0522 | 0.0585 |
|  | (0.326) | (0.223) | (0.235) |
| $10,000 - $19,999 | 0.0339 | 0.0647 | 0.0618 |
|  | (0.183) | (0.246) | (0.241) |
| $20,000 - $29,999 | 0.153 | 0.0827 | 0.0894 |
|  | (0.363) | (0.276) | (0.286) |
| $30,000 - $39,999 | 0 | 0.121 | 0.109 |
|  | (0) | (0.326) | (0.312) |
| $40,000 - $49,999 | 0.0847 | 0.0971 | 0.0959 |
|  | (0.281) | (0.296) | (0.295) |
| $50,000 - $59,999 | 0.153 | 0.0971 | 0.102 |
|  | (0.363) | (0.296) | (0.303) |
| $60,000 - $69,999 | 0.0678 | 0.0665 | 0.0667 |
|  | (0.254) | (0.249) | (0.250) |
| $70,000 - $79,999 | 0.153 | 0.0827 | 0.0894 |
|  | (0.363) | (0.276) | (0.286) |
| $80,000 - $89,999 | 0.0508 | 0.0450 | 0.0455 |
|  | (0.222) | (0.207) | (0.209) |
| $90,000 - $99,999 | 0.0169 | 0.0629 | 0.0585 |
|  | (0.130) | (0.243) | (0.235) |
| $100,000 - $124,999 | 0.0847 | 0.0809 | 0.0813 |
|  | (0.281) | (0.273) | (0.274) |
| $125,000 - $149,999 | 0.0339 | 0.0612 | 0.0585 |
|  | (0.183) | (0.240) | (0.235) |
| >$150,000 | 0.0508 | 0.0863 | 0.0829 |
|  | (0.222) | (0.281) | (0.276) |
| *Values here reflect proportions within the corresponding population.* | | | |
| **Employment status** |  |  |  |
| Employed for wage | 0.424 | 0.493 | 0.486 |
|  | (0.498) | (0.500) | (0.500) |
| Homemaker | 0.0508 | 0.0522 | 0.0520 |
|  | (0.222) | (0.223) | (0.222) |
| Military | 0 | 0.00360 | 0.00325 |
|  | (0) | (0.0599) | (0.0570) |
| Out of work and looking for work | 0.0339 | 0.0917 | 0.0862 |
|  | (0.183) | (0.289) | (0.281) |
| Out of work and not looking for work | 0.0339 | 0.0216 | 0.0228 |
|  | (0.183) | (0.145) | (0.149) |
| Retired | 0 | 0.0288 | 0.0260 |
|  | (0) | (0.167) | (0.159) |
| Self-employed | 0.339 | 0.129 | 0.150 |
|  | (0.477) | (0.336) | (0.357) |
| Student | 0.0847 | 0.158 | 0.151 |
|  | (0.281) | (0.365) | (0.359) |
| Unable to work | 0.0339 | 0.0216 | 0.0228 |
|  | (0.183) | (0.145) | (0.149) |
| *Values here reflect proportions within the corresponding population.* | | | |
| **Education Level** |  |  |  |
| Nursery school - 8th grade | 0 | 0.00180 | 0.00163 |
|  | (0) | (0.0424) | (0.0403) |
| Some highschool, no diploma | 0.0508 | 0.0180 | 0.0211 |
|  | (0.222) | (0.133) | (0.144) |
| High school graduate | 0.186 | 0.162 | 0.164 |
|  | (0.393) | (0.369) | (0.371) |
| Some college but no degree, have a diploma | 0.237 | 0.223 | 0.224 |
|  | (0.429) | (0.417) | (0.418) |
| College degree | 0.322 | 0.408 | 0.400 |
|  | (0.471) | (0.492) | (0.490) |
| Master's degree | 0.186 | 0.162 | 0.164 |
|  | (0.393) | (0.369) | (0.371) |
| Doctorate | 0.0169 | 0.0252 | 0.0244 |
|  | (0.130) | (0.157) | (0.154) |
| *Values here reflect proportions within the corresponding population.* | | | |
| **Political Affiliation** |  |  |  |
| Democrat | 0.0678 | 0.378 | 0.348 |
|  | (0.254) | (0.485) | (0.477) |
| Independent | 0.254 | 0.191 | 0.197 |
|  | (0.439) | (0.393) | (0.398) |
| Republican | 0.576 | 0.326 | 0.350 |
|  | (0.498) | (0.469) | (0.477) |
| Declined to answer | 0.102 | 0.106 | 0.106 |
|  | (0.305) | (0.308) | (0.308) |
| *Values here reflect proportions within the corresponding population.* | | | |
| **Political Conservativeness** |  |  |  |
| Degree of political conservativeness | 3.695 | 2.743 | 2.834 |
|  | (1.263) | (1.307) | (1.332) |
| *Values here reflect means of a likert scale from 1 to 5.* | | | |

Standard Deviation in parentheses.
