# Supplementary material for: When face masks signal social identity: Explaining the deep face-mask divide during the COVID-19 pandemic
Source: PLoS One. 2021 Jun 10;16(6):e0253195. doi: 10.1371/journal.pone.0253195 (PMC8191909; doi:10.1371/journal.pone.0253195)
Supplement: S2 Table — * 0.10 ** 0.05 *** 0.01. Standard errors in parentheses, clustered at individual level. Marginal effects from a Pooled Probit Regression using data on cooperation towards mask wearers, non-mask wearers and anonymous partners. For Panel A, marginal effects are relative to that of having an anonymous partner. For Panel B, marginal effects are relative to that of being a mask wearer. Includes controls for gender, age, ethnicity, the political party supported, education, household income, the exchange rate, and the order of the PD games. (DOCX) [file pone.0253195.s003.docx]

**S2 Table: Marginal effects within and across conditions**

| **Panel A: across partner mask wearing conditions** | | |
| --- | --- | --- |
| *Subgroup:* | Marginal effect of having a mask wearing partner | Marginal effect of having a non-mask wearing partner |
| *Non-Mask Wearers* | -0.138* | 0.032 |
|  | (0.074) | (0.068) |
| *Mask Wearers* | 0.074*** | -0.253*** |
|  | (0.019) | (0.025) |
| **Panel B: within partner mask wearing conditions** | | |
| *Subgroup:* | Marginal effect of  being a non-mask wearer | |
| *Random Partner* | -0.023 | |
|  | (0.065) | |
| *Mask Wearing Partner* | -0.234*** | |
|  | (0.069) | |
| *Non-Mask Wearing Partner* | 0.263*** | |
|  | (0.064) | |

* 0.10 ** 0.05 *** 0.01. Standard errors in parentheses, clustered at individual level. Marginal effects from a Pooled Probit Regression using data on cooperation towards mask wearers, non-mask wearers and anonymous partners. For Panel A, marginal effects are relative to that of having an anonymous partner. For Panel B, marginal effects are relative to that of being a mask wearer. Includes controls for gender, age, ethnicity, the political party supported, education, household income, the exchange rate, and the order of the PD games.
