# Supplementary material for: When face masks signal social identity: Explaining the deep face-mask divide during the COVID-19 pandemic
Source: PLoS One. 2021 Jun 10;16(6):e0253195. doi: 10.1371/journal.pone.0253195 (PMC8191909; doi:10.1371/journal.pone.0253195)
Supplement: S3 Table — * 0.10 ** 0.05 *** 0.01. Errors clustered at individual level. Marginal effects from a Pooled Probit Regression using data on cooperation towards mask wearers and non-mask wearers. Includes controls for gender, age, ethnicity, the political party supported, education, household income, the exchange rate, and the order of the PD games. (DOCX) [file pone.0253195.s004.docx]

**S3 Table: Mediators of cooperation**

| Variable: | Marginal effect  on cooperation |
| --- | --- |
| Beliefs about partner’s cooperation | 0.004*** |
|  | (0.001) |
| Beliefs about partner’s belief of own cooperation | 0.002*** |
|  | (0.001) |
| Altruism towards partner | 0.004*** |
|  | (0.000) |

* 0.10 ** 0.05 *** 0.01. Standard errors in parentheses, clustered at individual level. Marginal effects from a Pooled Probit Regression using data on cooperation towards mask wearers and non-mask wearers. Includes controls for gender, age, ethnicity, the political party supported, education, household income, the exchange rate, and the order of the PD games.
