# Supplementary material for: When face masks signal social identity: Explaining the deep face-mask divide during the COVID-19 pandemic
Source: PLoS One. 2021 Jun 10;16(6):e0253195. doi: 10.1371/journal.pone.0253195 (PMC8191909; doi:10.1371/journal.pone.0253195)
Supplement: S4 Table — * 0.10 ** 0.05 *** 0.01. OLS regressions with controls for own mask usage, gender, age, ethnicity, education, household income, the exchange rate, and the order of the PD games. Baseline group is Democrats. See S5 Table for more details. (DOCX) [file pone.0253195.s005.docx]

**S4 Table: Opinions on Covid-19 by Political Affiliation**

| Opinions on: | Scared  of Covid | Severity  of Covid | Guilt if Spread  Covid | Likelihood of infection: Self | Likelihood of infection: Neighbourhood | Likelihood of infection: State |
| --- | --- | --- | --- | --- | --- | --- |
| Independent | -0.754*** | -0.564*** | -0.194 | -0.276 | -0.264 | -0.251 |
|  | (0.219) | (0.166) | (0.171) | (0.193) | (0.174) | (0.168) |
| Republican | -0.863*** | -1.002*** | -0.404*** | -0.137 | -0.330** | -0.337** |
|  | (0.196) | (0.148) | (0.153) | (0.173) | (0.156) | (0.151) |
| Constant | 2.909 | 2.284 | 2.085 | 5.803*** | 4.054*** | 6.098*** |
|  | (1.950) | (1.474) | (1.519) | (1.717) | (1.546) | (1.494) |
| Observations | 615 | 615 | 615 | 615 | 615 | 615 |

* 0.10 ** 0.05 *** 0.01. Standard errors in parentheses. OLS regressions with controls for own mask usage, gender, age, ethnicity, education, household income, the exchange rate, and the order of the PD games. Baseline group is Democrats.
