# Supplementary material for: When face masks signal social identity: Explaining the deep face-mask divide during the COVID-19 pandemic
Source: PLoS One. 2021 Jun 10;16(6):e0253195. doi: 10.1371/journal.pone.0253195 (PMC8191909; doi:10.1371/journal.pone.0253195)
Supplement: S5 Table — * 0.10 ** 0.05 *** 0.01. OLS regressions with controls for own mask usage, gender, age, ethnicity, education, household income, the exchange rate, and the order of the PD games. Baseline group is Democrats. See S2 and S3 Tables for more details. (DOCX) [file pone.0253195.s006.docx]

**S5 Table: Opinions on Mask Wearing by Political Affiliation**

| Opinions on: | Use Frequency | Choice or Responsibility | Correct thing to do | Protects Self | Looks silly | Is ineffective | Will free ride | Not necessary |
| --- | --- | --- | --- | --- | --- | --- | --- | --- |
| Independent | -0.021 | -0.096** | -0.251** | -0.622*** | 0.357* | 0.738*** | 0.003 | 0.266* |
|  | (0.076) | (0.043) | (0.108) | (0.195) | (0.203) | (0.171) | (0.131) | (0.150) |
| Republican | -0.203*** | -0.135*** | -0.393*** | -0.659*** | 0.585*** | 1.068*** | 0.361*** | 0.569*** |
|  | (0.068) | (0.038) | (0.097) | (0.175) | (0.182) | (0.153) | (0.117) | (0.135) |
| Constant | 2.162*** | 1.362*** | 2.949*** | 3.629** | 5.213*** | 5.715*** | 7.122*** | 8.484*** |
|  | (0.679) | (0.380) | (0.961) | (1.734) | (1.804) | (1.524) | (1.162) | (1.338) |
| Observations | 615 | 615 | 615 | 615 | 615 | 615 | 615 | 615 |

* 0.10 ** 0.05 *** 0.01. Standard errors in parentheses. OLS regressions with controls for own mask usage, gender, age, ethnicity, education, household income, the exchange rate, and the order of the PD games. Baseline group is Democrats.
