# Supplementary material for: When face masks signal social identity: Explaining the deep face-mask divide during the COVID-19 pandemic
Source: PLoS One. 2021 Jun 10;16(6):e0253195. doi: 10.1371/journal.pone.0253195 (PMC8191909; doi:10.1371/journal.pone.0253195)
Supplement: S6 Table — * 0.10 ** 0.05 *** 0.01. OLS regressions with controls for own mask usage, gender, age, ethnicity, education, household income, the exchange rate, and the order of the PD games. Baseline group is Democrats. See S4 Table for more details. (DOCX) [file pone.0253195.s007.docx]

**S6 Table: Relative Moral Opinions by Political Affiliation**

|  | Opinion on Mask wearers relative to Non-Mask Wearers for | | | | | |
| --- | --- | --- | --- | --- | --- | --- |
|  | Care | Fairness | Authority | Loyalty | Sanctity | Liberty |
| Independent | -0.539** | -0.420 | -0.511* | -0.426 | -0.276 | -0.102 |
|  | (0.240) | (0.263) | (0.260) | (0.274) | (0.279) | (0.288) |
| Republican | -0.930*** | -0.782*** | -0.716*** | -0.331 | -0.768*** | -0.161 |
|  | (0.215) | (0.236) | (0.233) | (0.246) | (0.250) | (0.258) |
| Constant | -3.650* | -1.986 | 1.178 | -4.332* | -2.707 | -2.358 |
|  | (2.137) | (2.340) | (2.313) | (2.439) | (2.486) | (2.559) |
| Observations | 615 | 615 | 615 | 615 | 615 | 615 |

* 0.10 ** 0.05 *** 0.01. Standard errors in parentheses. OLS regressions with controls for own mask usage, gender, age, ethnicity, education, household income, the exchange rate, and the order of the PD games. Baseline group is Democrats.
