# Supplementary material for: When face masks signal social identity: Explaining the deep face-mask divide during the COVID-19 pandemic
Source: PLoS One. 2021 Jun 10;16(6):e0253195. doi: 10.1371/journal.pone.0253195 (PMC8191909; doi:10.1371/journal.pone.0253195)
Supplement: S7 Table — * 0.10 ** 0.05 *** 0.01. Errors clustered at individual level. Marginal effects from a Pooled Probit Regression using data on cooperation towards mask wearers, non-mask wearers and anonymous partners. Regressions in Panel A control for the interaction between political party and mask wearing partner; marginal effects are relative to that of being a Democrat. Regressions in Panel B control for the interaction between conservativeness and mask wearing partner. Also includes controls for gender, age, ethnicity, the political party supported, education, household income, the exchange rate, and the order of the PD games. (DOCX) [file pone.0253195.s008.docx]

**S7 Table: Marginal effects of Political Variables**

| **Panel A: Political Affiliation** | | |
| --- | --- | --- |
| *Subgroup:* | Marginal effect of being Independent | Marginal effect of being Republican |
| *Mask Wearing Partner* | -0.049 | -0.103** |
|  | (0.051) | (0.044) |
| *Non-Mask Wearing Partner* | 0.083 | 0.080 |
|  | (0.058) | (0.050) |
|  |  |  |
| **Panel B: Conservativeness** | | |
| *Subgroup:* | Marginal effect of  being more Conservative | |
| *Mask Wearing Partner* | -0.044*** | |
|  | (0.014) | |
| *Non-Mask Wearing Partner* | 0.009 | |
|  | (0.015) | |
|  |  | |

* 0.10 ** 0.05 *** 0.01. Standard errors in parentheses, clustered at individual level. Marginal effects from a Pooled Probit Regression using data on cooperation towards mask wearers, non-mask wearers and anonymous partners. Regressions in Panel A control for the interaction between political party and mask wearing partner; marginal effects are relative to that of being a Democrat. Regressions in Panel B control for the interaction between conservativeness and mask wearing partner. Also includes controls for gender, age, ethnicity, the political party supported, education, household income, the exchange rate, and the order of the PD games.
