# Supplementary material for: When face masks signal social identity: Explaining the deep face-mask divide during the COVID-19 pandemic
Source: PLoS One. 2021 Jun 10;16(6):e0253195. doi: 10.1371/journal.pone.0253195 (PMC8191909; doi:10.1371/journal.pone.0253195)
Supplement: S8 Table — * 0.10 ** 0.05 *** 0.01. Errors clustered at individual level. OLS regressions using data on expectations and altruism towards mask wearers and non-mask wearers. Includes controls for gender, age, ethnicity, education, household income, the session, and the order of the PD games. (DOCX) [file pone.0253195.s009.docx]

**S8 Table: Interaction between Political Affiliation and Partner Type**

**on Mediators of Cooperation**

|  | Altruism | Beliefs about | Beliefs about |
| --- | --- | --- | --- |
|  | Towards  partner | Partner’s  Cooperation | Partner’s Beliefs about own Cooperation |
| Mask-wearing Partner | 35.930*** | 26.453*** | 17.383*** |
|  | (2.161) | (1.772) | (1.878) |
| Mask-wearing Partner $\times$ Independent | -3.250 | -7.270** | -5.152* |
|  | (2.329) | (3.012) | (3.040) |
| Mask-wearing Partner $\times$ Republican Party | -5.363*** | -7.540*** | -5.437** |
|  | (1.965) | (2.755) | (2.717) |
| Non-Mask-wearing Partner # Independent | 5.102** | 0.737 | -3.273 |
|  | (2.498) | (2.987) | (3.215) |
| Non-Mask-wearing Partner # Republican Party | 7.665*** | 8.567*** | 2.095 |
|  | (2.111) | (2.609) | (2.851) |
| Mask-wearer | -5.667*** | 4.704* | 8.298*** |
|  | (1.779) | (2.768) | (3.083) |
| Constant | 43.783*** | 20.770*** | 44.543*** |
|  | (3.800) | (6.677) | (7.201) |
| Observations | 1230 | 1230 | 1230 |

* 0.10 ** 0.05 *** 0.01. Standard errors in parentheses, clustered at individual level. OLS regressions using data on expectations and altruism towards mask wearers and non-mask wearers. Includes controls for gender, age, ethnicity, education, household income, the session, and the order of the PD games.
