# Supplementary material for: When face masks signal social identity: Explaining the deep face-mask divide during the COVID-19 pandemic
Source: PLoS One. 2021 Jun 10;16(6):e0253195. doi: 10.1371/journal.pone.0253195 (PMC8191909; doi:10.1371/journal.pone.0253195)
Supplement: S9 Table — * 0.10 ** 0.05 *** 0.01. Errors clustered at individual level. OLS regressions using data on expectations and altruism towards mask wearers and non-mask wearers. Includes controls for gender, age, ethnicity, education, household income, the session, and the order of the PD games. (DOCX) [file pone.0253195.s010.docx]

**S9 Table: Interaction between Political Conservativeness and Partner Type**

**on Mediators of Cooperation**

|  | Altruism | Beliefs about | Beliefs about |
| --- | --- | --- | --- |
|  | Towards  partner | Partner’s  Cooperation | Partner’s Beliefs about own Cooperation |
| Mask-wearing Partner | 45.424*** | 35.018*** | 20.237*** |
|  | (3.414) | (2.801) | (2.758) |
| Mask-wearing Partner $\times$ conservativeness | -2.513*** | -3.002*** | -2.172** |
|  | (0.670) | (0.869) | (0.855) |
| Non-Mask-wearing Partner $\times$conservativeness | 3.251*** | 2.740*** | 0.067 |
|  | (0.711) | (0.821) | (0.884) |
| Mask-wearer | -5.656*** | 4.669 | 8.018** |
|  | (1.812) | (2.844) | (3.131) |
| Constant | 38.803*** | 13.027* | 41.225*** |
|  | (4.095) | (6.845) | (7.325) |
| Observations | 1230 | 1230 | 1230 |

* 0.10 ** 0.05 *** 0.01. Standard errors in parentheses, clustered at individual level. OLS regressions using data on expectations and altruism towards mask wearers and non-mask wearers. Includes controls for gender, age, ethnicity, education, household income, the session, and the order of the PD games.
