# Supplementary material for: When face masks signal social identity: Explaining the deep face-mask divide during the COVID-19 pandemic
Source: PLoS One. 2021 Jun 10;16(6):e0253195. doi: 10.1371/journal.pone.0253195 (PMC8191909; doi:10.1371/journal.pone.0253195)
Supplement: S10 Table — * 0.10 ** 0.05 *** 0.01. Errors clustered at individual level. Marginal effects from a Pooled Probit Regression using data on cooperation towards mask wearers, non-mask wearers and anonymous partners. Regressions control for the interaction between political party and mask wearing partner, and the interaction between own mask usage and mask wearing partner. Marginal effects are relative to that of being mask wearer. Also, includes controls for gender, age, ethnicity, the political party supported, education, household income, the session, and the order of the PD games. (DOCX) [file pone.0253195.s011.docx]

**S10 Table: Marginal Effects within partner mask wearing conditions**

| *Subgroup:* | Marginal effect of  being a non-mask wearer |
| --- | --- |
| *Random Partner* | -0.025 |
|  | (0.065) |
| *Mask Wearing Partner* | -0.212*** |
|  | (0.069) |
| *Non-Mask Wearing Partner* | 0.246*** |
|  | (0.065) |

* 0.10 ** 0.05 *** 0.01 Standard errors in parentheses, clustered at individual level. Marginal effects from a Pooled Probit Regression using data on cooperation towards mask wearers, non-mask wearers and anonymous partners. Regressions control for the interaction between political party and mask wearing partner, *and the interaction between own mask usage and mask wearing partner.* Marginal effects are relative to that of being mask wearer. Also, includes controls for gender, age, ethnicity, the political party supported, education, household income, the session, and the order of the PD games.
